# A multilayer temporal network model for STD spreading accounting for permanent and casual partners

**DOI:** 10.1038/s41598-020-60790-0

**Published:** 2020-03-02

**Authors:** Aram Vajdi, David Juher, Joan Saldaña, Caterina Scoglio

**Affiliations:** 1 Kansas StateUniversity, Department of Electrical and Computer Engineering, Manhattan, Kansas USA; 2 Universitat de Girona, Department of Computer Science, Applied Mathematics, and Statistics, Girona, Catalonia Spain

**Keywords:** Biophysics, Mathematics and computing

## Abstract

Sexually transmitted diseases (STD) modeling has used contact networks to study the spreading of pathogens. Recent findings have stressed the increasing role of casual partners, often enabled by online dating applications. We study the Susceptible-Infected-Susceptible (SIS) epidemic model –appropriate for STDs– over a two-layer network aimed to account for the effect of casual partners in the spreading of STDs. In this novel model, individuals have a set of steady partnerships (links in layer 1). At certain rates, every individual can switch between active and inactive states and, while active, it establishes casual partnerships with some probability with active neighbors in layer 2 (whose links can be thought as potential casual partnerships). Individuals that are not engaged in casual partnerships are classified as inactive, and the transitions between active and inactive states are independent of their infectious state. We use mean-field equations as well as stochastic simulations to derive the epidemic threshold, which decreases substantially with the addition of the second layer. Interestingly, for a given expected number of casual partnerships, which depends on the probabilities of being active, this threshold turns out to depend on the duration of casual partnerships: the longer they are, the lower the threshold.

## Introduction

The rising number of infected individuals with sexually transmitted diseases (STD) is a significant concern for public health. It is estimated there are one million new cases of curable STDs acquired each day globally^1^. Specifically, the reports from the Centers for Disease Control and Prevention (CDC) in the U.S. show a dramatic increase in new infections from chlamydia, gonorrhea, and syphilis since 2013. Although common STDs can be treated with antibiotics, antibiotic resistance can exacerbate the situation. It is well known that the structure of the sexual network plays a major role in the spread of STDs^2,3^. Indeed, with the increasing trend in online dating, sexual networks become more complex and dynamic. For example, a recent study indicates a relationship between using an online dating application and having had five or more previous sexual partners in young adults^4^. To capture the effect of the sexual network and pair formation on the spread of STDs, researchers have developed various mathematical models. However, these models do not consider the heterogeneity in link formation at the individual level because their formulation is based on a mean-field description at the level of pairs^5,6^ or on a statistical description of the sexual network^7^. Individual-based stochastic models have been traditionally developed when a more detailed description of individuals is considered in pair formation, including individual age, different infectious periods, and concurrent partnerships^3,8,9^.

In this paper, we develop a model that incorporates the effect of each individual in the sexual network on the spread of STDs. The susceptible-infected-susceptible (SIS) model over a complex network is a mathematical approach for describing the spread of a pathogen in a population with heterogeneous connectivity among individuals^10–13^. In this context, a stochastic approach is suitable because the description of the spreading process includes a degree of uncertainty at the individual level that is not present when one assumes fully mixing among members of the populations (compartmental models). Such an uncertainty can be quantified using stochastic individual-based models to obtain a distribution of outcomes. Indeed, the analysis of the SIS model over static networks has clarified the role of network structure in the emergence of the endemic state^3^. This result, in turn, has provided opportunities to control an epidemic by altering the network structure, even though there are multiple sources of uncertainty at the individual level^14–16^.

In the SIS epidemic model on networks, individuals are represented by nodes that are either susceptible or infected. If a node is susceptible, it becomes infected due to interactions with its infected neighbors, and if it is infected, it can recover and become susceptible again. Although there are articles that consider non-Markovian spreading processes^17,18^, the most common assumption in the networked spreading literature is that the time duration a node stays infected is a random variable with an exponential distribution. Moreover, the same assumption is held for the infection process. In other words, the probability that a susceptible node with one infected neighbor stays susceptible decreases exponentially with time. An important result regarding the SIS model is that the infection in a population dies out exponentially fast if *β*/*δ* < 1/*λ*_*max*_(*A*) where *β* and *δ* are the infection transmission and recovery rates, and *λ*_*max*_(*A*) is the largest eigenvalue of the static network adjacency matrix^10,11^. This result is obtained using the N-intertwined equation which approximately describes the SIS model, whose exact mathematical treatment is unfeasible^19^. In fact, it is shown that the N-intertwined equations provide an upper bound for the prevalence of infection in the exact SIS process^20,21^. Although the aforementioned result is significant for controlling epidemic, the assumption that the underlying network is known and static is not justifiable in some important instances of real-world populations. For example, casual partnerships resulting from the current trend in online dating cannot be represented as a static network. This motivates our work to analyze the SIS spreading process over networks with time-varying aspects in their architecture.

In fact, in the existing literature, we can find several works analyzing the SIS processes over various models of dynamic networks^22–24^. Paré *et al*. analyze the N-intertwined approximation of the SIS process when the adjacency matrix of the network is a deterministic and continuous function of time^25^. Another approach to model temporariness of partnerships is to adopt the switching network concept. In such a model the contact network randomly switches among a set of predetermined adjacency matrices. In^26,27^ the authors have studied sufficient conditions for the stability of the disease-free equilibrium in the SIS spreading model over switching networks. In^28^ the authors analyze the initial phase of an SIS epidemic on a network with preventive rewiring. Another class of time-varying network that has been studied in the existing literature is the edge-Markovian networks where the edges appear and disappear following independent Markov processes^29^. In^30^, the authors have used an improved effective degree compartmental modeling framework to study the SIS spreading process in the edge-Markovian networks. Ogura *et al*. consider a generalized version of edge-Markovian model where the inter-event time distribution for the appearance and disappearance of the links is not necessarily exponential^31^. Moreover, they provide a sufficient condition for the exponential stability of the disease-free state in the SIS process that is unfolding on such a time-varying network. A different approach to model time-varying networks are the so-called activity-driven network models which have been studied mostly in the physics literature^32–34^. In these models, the probability that an individual engages in a connection with other individuals is determined by a random variable called "activity”. Typically, in a discrete-time activity-driven model (see^32^ and, for multiplex networks,^34^), at each time step all the existing links are deleted and each node becomes active according to this activation probability. Then, if active, a node establishes links with randomly selected nodes (active or not) in the population. Certainly, in some practical cases most of the previous models are oversimplifications of the real scenario and it may miss some critical aspects for the infection spreading in a population.

The goal of this paper is to assess the importance of casual partnerships in the propagation of STDs. In the study and control of this type of infections, the importance of the sexual networks has always been recognized^2^. Many public health departments have partner-notification programs aimed to discover the network of sexual relationships among members of a community in order to seek evaluation of exposed partners and treatment of confirmed cases^35^. However, it is known that there are partners who are not disclosed during the contact tracing because of sampling biases in data collection, the existence of untraceable contacts or dead-ends, or simply because participants do not reveal all of their sexual partners^36^. The typical notification pattern found in^37^ was to notify a main partner but not others. Similarly, in^38^ partner notification was found to be less effective for casual and short term (<7 days) partnerships. So, casual partners are less likely to be notified and, hence, they may move on to infect new partners. According to these observations, we will consider the existence of a set of disclosed partners that we identify as steady or long-lasting partnerships, and a set of undisclosed ones that we identify as casual partners. The set of steady partnerships defines the first layer of the sexual network.We assume that steady partnerships last several infectious periods of the disease under consideration.

The set of potential casual partnerships defines the second layer of the network where nodes are individuals and links represent potential casual partnerships for each individual. In this setting, an individual (node) can become *active* at a given rate and establish casual partnerships among other individuals (nodes) that have also become active. So, we combine the idea of having different neighborhoods which underlies switching network models, and that of nodal activity present in activity driven models. However, in contrast to these approaches, here we are not assuming a reshuffling of neighbours at each time step^34^ or a sequential switching of network architectures^27^. Instead we assume that, each time a node becomes active, a subset of casual partnerships is randomly chosen among the set of potential casual partnerships (potential links) defined by the second layer. Precisely, a potential link can become a casual partnership with a link-specific probability when the nodes at its both ends are active, and it remains so until one of the nodes becomes inactive again (end of the casual partnership). Within each type of partnership (steady and casual), sexual contact is assumed to occur on a regular basis and the risk of transmission is constant per contact.

The role of casual partnerships in the incidence of STDs has been also considered in pair formation models^5,6,39,40^. In classic pair formation models the members of a population are classified as singles or in partnership. The latter belong to pairs characterized by a low separation rate. Both, singles and pairs, are involved in casual partnerships, i.e. pairs of very short duration. In these models, casual pairs are simply modeled as instantaneous contacts in such a way that they are considered only in terms accounting for new infections in the corresponding equations^5,39,40^. A pair formation model with true compartments for casual partnerships has been considered in^6,39^ where a given fraction of newly created partnerships are casual whereas the remaining ones are steady. The difference between them are the values of the separation rates assigned to each type of pair. Moreover, a one-layer dynamic sexual network model has been introduced in^41^. In this model, casual partnerships are considered again as instantaneous encounters and, in its deterministic formulation, casual partnerships appear as in classic pair formation models. Here, we combine a sexual network description of steady and casual partnerships with a dynamical model for these casual partnerships.

The temporary character of sexual network results from the transitions between active and inactive states. The idea is that, each time a node becomes active, a new search for new casual partnerships occurs among the set of potential partnerships defined by the second network layer. As far as we know, the impact of such transitions on the spread of an epidemic has been neglected in previous models. For instance, in activity-driven models the continuous reshuffling of neighbors in each layer and the way new contacts are created forbid an analysis as the one we present here. In particular, we address the question whether highly frequent casual partnerships of short duration have the same impact on the disease prevalence as less frequent casual partnerships of longer duration. A first guess could be that, as long as the probability of being active is the same in both cases, the impact should be the same no matter how a node visits the layer of casual partners. However, as we will see, this is not the correct answer.

In summary, we make the following contributions:

- We propose a new framework for the modeling of temporal networks that can capture the heterogeneity of randomness in developing the casual partnerships among different nodes. Moreover, our model can capture the effect of the duration of casual partnerships in STD spreading.
- We develop a mean-field approximation to describe the SIS spreading process over such a temporal network. We also discuss why such an approximation is relevant to the exact description of the process.
- We analyze the disease-free state of the SIS spreading process using the mean-field equations and we find a condition that guarantees the exponential die out of an infection in a time-varying network that can be described via our modeling approach.
- Using the exact simulation of the process, we show how the duration of casual partnerships can affect the meta-stable state of the SIS spreading process.

In the next section, we develop a temporal network model and a mean-field type approximation to describe the SIS spreading process over such a temporal network. In sections 3, we analyze the disease-free state of the SIS spreading process using the mean-field equations and we find a condition that guarantees the exponential die out of an infection in a time-varying network that can be described via our modeling approach. Finally, using the exact simulation of the process, in section 4 we show how the duration of casual partnerships can affect the metastable state of the SIS spreading process.

## The model

In the following, we first introduce the notation and the assumptions of the two-layer temporal network model, and later, we develop the SIS mean field-equations on this network model.

### Two-layer temporal network model

**Figure 1:**
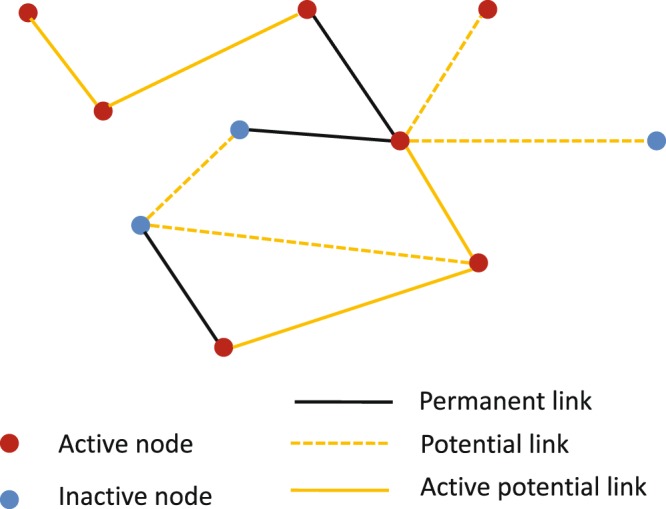
A snapshot from a realization of the network model. At any time *t* the nodes are either active or inactive. A potential link is activated with probability *p*_0_ if its both ends are active at the same time.

We consider a population of *N* agents that are connected with two different types of links. The first network layer, ${{\mathbb{L}}}_{1}$, represents steady partnerships among the agents. Independently of the relationship paradigm one wants to account for (serial monogamy, polygamy, etc.), it is reasonable to expect that ${{\mathbb{L}}}_{1}$ is a quite sparse disconnected network, specially when considering a time scale of interest for SIS-type infections. Beside these permanent links, we assume a second type of links that correspond to potential casual partnerships. These links become active with a probability *p*_0_ only when the agents at both ends of the links are simultaneously seeking casual partners. This second layer of links is denoted by ${{\mathbb{L}}}_{2}$. In general, *p*_0_ can be different for each pair of nodes. However, for clarity of the presentation and since it is straightforward to generalize our result to the heterogeneous case, we assume the same *p*_0_ value for all potential links. By definition, the intersection of the sets of links in ${{\mathbb{L}}}_{1}$ and ${{\mathbb{L}}}_{2}$ is empty. In contrast to the low connectivity of ${{\mathbb{L}}}_{1}$, it is plausible, especially from the advent of match-making apps outside social networks, that ${{\mathbb{L}}}_{2}$ is highly connected. So, we will assume that ${{\mathbb{L}}}_{2}$ is connected (that is, for any pair {*i*, *j*} of nodes there is a path of links in ${{\mathbb{L}}}_{2}$ joining *i* and *j*). While the links in layer ${{\mathbb{L}}}_{1}$ always can transmit infection, a link in layer ${{\mathbb{L}}}_{2}$ transmits the infection only when it becomes an active link. In the model the activation of a potential link in ${{\mathbb{L}}}_{2}$ depends on the activity state of agents at both ends of the link. Apart from the node infection state, we assume that nodes are either active or inactive at any time *t*. When a node becomes active, it seeks a partner among its active neighbors in ${{\mathbb{L}}}_{2}$ and, with a probability *p*_0_, it is established a casual partnership. Later, when one of the two nodes goes to the inactive state, the link is inactivated. This node transition between active and inactive states introduces temporariness in the sexual network. Here, we assume node activation processes are independent Poisson processes, where node *i* becomes active with rate ${\gamma }_{1}^{i}$, and if it is active, it goes to the inactive state with rate ${\gamma }_{2}^{i}$. Since the inverse of the transition rate is the expected value of transition time, if node *i* is active, it is expected to stay active for a period of time of length ${({\gamma }_{2}^{i})}^{-1}$. Thus, when we want to model a node that is frequently activating occasional links, we can assign high values of *γ*_2_ and *γ*_1_ to that node. Moreover, if a node does not participate in casual partnerships —it never becomes active— *γ*_1_ is set equal to zero for that node. Figure 1 shows a snapshot of a realization of the temporal network.

Since the inactivation time for each node has an exponential distribution, and the inactivation of a temporal link depends on its both ends, it is straightforward to see that the duration of a casual partnership has an exponential distribution. In fact, a temporal link disappears when one of its ends becomes inactive. Since the minimum of two independent random variables with exponential distributions is distributed exponentially with a rate that is summation of the rates in the independent distributions, it follows that the duration of a temporal link between nodes *i* and *j* has an exponential distribution with the rate ${\gamma }_{2}^{i}+{\gamma }_{2}^{j}$. Hence, the expected duration of a casual partnership is ${T}_{i,j}={({\gamma }_{2}^{i}+{\gamma }_{2}^{j})}^{-1}$. Moreover, a straightforward calculation shows that, after some initial period of time, the probability to find node *i* in the active state is ${p}_{2}^{i}={\gamma }_{1}^{i}/({\gamma }_{2}^{i}+{\gamma }_{1}^{i})$. Hence, in the steady state of the process, the probability for the existence of a casual link between the two nodes is ${P}_{i,j}={p}_{2}^{i}{p}_{2}^{j}{p}_{0}$.

Another describing measure of the network dynamics is the probability of link establishment between nodes *i* and *j* while *i* is active (assuming they are not connected to each other in ${{\mathbb{L}}}_{1}$). In the supplementary material, section 1, we have shown this probability can be written as

1 $${P}_{j| i}=\frac{{p}_{2}^{j}{p}_{0}}{1-{f}_{1}{f}_{2}(1-{p}_{0})}\left(1+\frac{{p}_{1}^{j}}{{p}_{2}^{j}}{f}_{2}\right),$$

where ${f}_{2}={\gamma }_{1}^{j}/({\gamma }_{2}^{i}+{\gamma }_{1}^{j})$, ${f}_{1}={\gamma }_{2}^{j}/({\gamma }_{2}^{i}+{\gamma }_{2}^{j})$ and ${p}_{1}^{j}={\gamma }_{2}^{j}/({\gamma }_{2}^{j}+{\gamma }_{1}^{j})$. From these expression of *P*_*j*ǀ*i*_, we can easily determine the average number of links *l*^*i*^ that node *i* develops every time it becomes active, namely, *l*^*i*^ = ∑_*j*_*P*_*j*ǀ*i*_. As an example, consider a population where each node has *k*_2_ potential links in ${{\mathbb{L}}}_{2}$ and the values of ${\gamma }_{2}^{i}$ and ${\gamma }_{1}^{i}$ are the same for all the nodes (${\gamma }_{2}^{i}={\gamma }_{2}$ and ${\gamma }_{1}^{i}={\gamma }_{1}$ ∀ *i*). In this case, the duration of temporal links are distributed exponentially with the average value of ${(2{\gamma }_{2})}^{-1}$. Moreover, the average number of active links for any node *i* during its active period becomes

2 $${l}^{i}=\frac{{k}_{2}{p}_{0}{p}_{2}(1+{p}_{1})}{1-0.5{p}_{2}(1-{p}_{0})},$$

where we have used that *f*_2_ = *p*_2_ when the nodes have the same *γ*_1_ and *γ*_2_. 

### SIS epidemics on two-layer temporal networks

In this section we develop a mean-field type approximation to describe the spreading of infection on the temporal network introduced in section 2.1. Next, we discuss the relevance of such an approximation to the exact spreading process.

The susceptible-infected-susceptible (SIS) model has been adopted for studying several STDs because it assumes no immunity after recovering from infection and, hence, it allows for multiple re-infections. This is the case, for instance, for Chlamydia and gonorrhoea where little or no immunity is acquired after infection^2,5^. In this model, each node is either susceptible (S) or infectious (I). We assume the infection and recovery processes are Poisson processes, where an infectious node recovers at a rate *δ* and transmits the infection to a susceptible neighbor at a rate *β*. When a susceptible node is in contact with several infectious nodes, it is assumed each infected neighbor acts independently. Thus, the susceptible node contracts the infection with a rate that is the sum of the rates of all the independent infection processes.

Combining the network model and the SIS spreading process, we deduce that each node can assume one of four different states: ${{\mathcal{S}}}_{1}$ susceptible and inactive, ${{\mathcal{S}}}_{2}$ susceptible and active, ${{\mathcal{I}}}_{1}$ infectious and inactive, ${{\mathcal{I}}}_{2}$ infectious and active. If ${S}_{1}^{i}$, ${S}_{2}^{i}$, ${I}_{1}^{i}$ and ${I}_{2}^{i}$ represent the probabilities that the node *i* is in one of the four states in the mean-field approximation, the equations for the time evolution of ${S}_{1}^{i}$, ${S}_{2}^{i}$, ${I}_{1}^{i}$ and ${I}_{2}^{i}$ can be written as

3a $${{\dot{S}}_{1}}^{i}=-{\gamma }_{1}^{i}{S}_{1}^{i}+{\gamma }_{2}^{i}{S}_{2}^{i}+\delta {I}_{1}^{i}-\beta \sum _{j}{a}_{1}^{ij}{S}_{1}^{i}({I}_{1}^{j}+{I}_{2}^{j}),$$

3b $${{\dot{I}}_{1}}^{i}=-{\gamma }_{1}^{i}{I}_{1}^{i}+{\gamma }_{2}^{i}{I}_{2}^{i}-\delta {I}_{1}^{i}+\beta \sum _{j}{a}_{1}^{ij}{S}_{1}^{i}({I}_{1}^{j}+{I}_{2}^{j}),$$

3c $$\begin{array}{lll}{{\dot{S}}_{2}}^{i} & = & -{\gamma }_{2}^{i}{S}_{2}^{i}+{\gamma }_{1}^{i}{S}_{1}^{i}+\delta {I}_{2}^{i}-\beta \sum _{j}{a}_{1}^{ij}{S}_{2}^{i}({I}_{1}^{j}+{I}_{2}^{j})\\  &  & -{\beta }^{{\prime} }\sum _{j}{a}_{2}^{ij}{S}_{2}^{i}{I}_{2}^{j},\end{array}$$

3d $$\begin{array}{lll}{{\dot{I}}_{2}}^{i} & = & -{\gamma }_{2}^{i}{I}_{2}^{i}+{\gamma }_{1}^{i}{I}_{1}^{i}-\delta {I}_{2}^{i}+\beta \sum _{j}{a}_{1}^{ij}{S}_{2}^{i}({I}_{1}^{j}+{I}_{2}^{j})\\  &  & +\,{\beta }^{{\prime} }\sum _{j}{a}_{2}^{ij}{S}_{2}^{i}{I}_{2}^{j},\end{array}$$

where ${\beta }^{{\prime} }={p}_{0}\beta$. In the equations above, ${a}_{1}^{ij}$ is an element of the adjacency matrix *A*_1_ for layer ${{\mathbb{L}}}_{1}$ with ${a}_{1}^{ij}=1$, if the nodes *i* and *j* form a steady partnership, and ${a}_{1}^{ij}=0$ otherwise. Similarly, ${a}_{2}^{ij}$ is the (*i*, *j*) element of the adjacency matrix *A*_2_ corresponding to layer ${{\mathbb{L}}}_{2}$. It is important to note that, when *p*_0_ has different values for each pair, we can absorb *p*_0_ in the adjacency matrix *A*_2_ and the elements of the *A*_2_ become the pair-specific probabilities of developing casual partnerships.

The first term on the r.h.s. of equation 3a reflects the fact that the inactive susceptible node *i* becomes active with a rate ${\gamma }_{1}^{i}$ and the second term indicates if the node *i* is in the state ${{\mathcal{S}}}_{2}$ it goes to the inactive state with a rate ${\gamma }_{2}^{i}$. The third term originates from the recovering process of inactive infected nodes. In the forth term, each addend is the multiplication of the probability that the node *i* is inactive susceptible and the probability that a permanent neighbor of node *i* is infected.

In equation 3c, we take into account the two different sets of neighbors that propagate infection to the active susceptible node *i*. The forth term on the r.h.s. arises from the contagion propagation by infectious steady partners of node *i*. In the fifth term, every summand is the multiplication of the probability that the node *i* is in the state ${{\mathcal{S}}}_{2}$ and the probability that a potential neighbor of node *i* in the activity layer ${{\mathbb{L}}}_{2}$ is infectious and also active. When the nodes *i* and *j* are active and they are neighbors in ${{\mathbb{L}}}_{2}$, they develop a link with probability *p*_0_. Hence, the summation in the fifth term of this equation is multiplied by *p*_0_.

**Figure 2:**
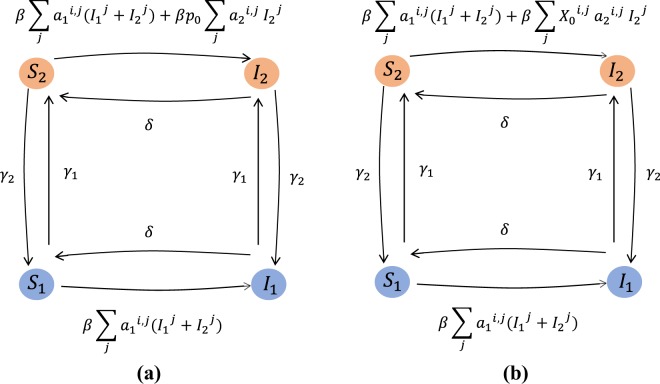
The figures show the diagrams of node transitions among different node states. The rate of each transition is specified on the arrow that indicates the transition. (**a**) shows diagram of the Markov process which is discussed in section 2.2, and (**b**) shows diagram of the exact process. In these figures ${I}_{1}^{j}=1$ (${I}_{2}^{j}=1$) if node *j* is infected and inactive (active), otherwise it is zero. In diagram (**b**) ${X}_{0}^{i,j}$ is a Bernoulli random variable that has value one with probability *p*_0_. This random variable is drawn each time a pair of active nodes (*i*, *j*) with a potential link between them occurs, regardless of their disease status.

Equations 3 describe approximately the (stochastic) spreading model whose exact mathematical description requires tracking the probability of the system being in any of 4^*N*^ possible states which is intractable. Our numerical simulations show these approximate equations lead to nodal infection probabilities that are upper bounds for the infection probabilities in the exact spreading model. In the following, we give an intuitive picture to justify the result of our simulations. Readers familiar with continuous-time Markov chain and the mean-field approximation of SIS process over static one-layer network^10^ may recognize that the equations 3 are the N-intertwined approximation of a continuous Markov process similar to our model but with a difference. In contrast to the exact description of our model, for this Markov process a link in layer ${{\mathbb{L}}}_{2}$ is activated whenever the nodes at both ends of the link are active with the infection transmission through the link being ${\beta }^{{\prime} }={p}_{0}\beta$ instead of *β*. Figure 2a shows the nodal transitions in the Markov process. Instead, in our model, when both ends of a link are active, the link becomes activated with probability *p*_0_, and transmits infection with rate *β* if one of the nodes is infected. Figure 2b shows the nodal transitions in our model. Our simulations show that the equations 3 give an upper bound for the nodal infection probabilities in the Markov process described above which, in turn, are higher than those of our stochastic model. To explain these results we invoke the argument in^20^, where the authors show the Markovian SIS process over a static one-layer network is upper bounded by the N-intertwined approximation. In fact, equation 3b would be an exact equation for the Markov process if we replace in this equation ${S}_{1}^{i}({I}_{1}^{j}+{I}_{2}^{j})$ with $\Pr ({x}_{i}={{\mathcal{S}}}_{1},{x}_{j}={{\mathcal{I}}}_{1}\,{\rm{or}}\,{{\mathcal{I}}}_{2})$, which is the joint probability that node *i* is inactive and susceptible, and node *j* is infected. Moreover, since two neighboring nodes can only enhance the infection probabilities of each other and their activity states are independent, the infection states would be non-negatively correlated. In other words, when we know node *j* is infected the expectation to observe node *i* in the susceptible state is less than the case when we do not know the state of node *j*,

$$\Pr ({x}_{i}={{\mathcal{S}}}_{1}| {x}_{j}={{\mathcal{I}}}_{1}\,{\rm{or}}\,{{\mathcal{I}}}_{2})\le \Pr ({x}_{i}={{\mathcal{S}}}_{1}).$$

If we rewrite the inequality above as

$$\Pr ({x}_{i}={{\mathcal{S}}}_{1},{x}_{j}={{\mathcal{I}}}_{1}\,{\rm{or}}\,{{\mathcal{I}}}_{2})\le {S}_{1}^{i}({I}_{1}^{j}+{I}_{2}^{j}),$$

we can see the addends in equation 3b are upper bounds for the corresponding terms, $\Pr ({x}_{i}={{\mathcal{S}}}_{1},{x}_{j}={{\mathcal{I}}}_{1}\,{\rm{or}}\,{{\mathcal{I}}}_{2})$, in the exact equation for the Markov process. Since these terms appears with positive sign, they only increase the infection probability. Using the same argument about the correlation of nodal infection in equation 3d, we expect the N-intertwined approximation in equation 3 gives an upper bound for the nodal infection probabilities in the Markov process and our simulations show that it is in fact an upper bound. In order to compare the nodal infection probabilities in the Markov model and the exact description of our stochastic model, consider an instance where at time *t*_1_ one end of an ${{\mathbb{L}}}_{2}$ link is active susceptible while the other end is active infected. If *t*_2_ is the later instant when either the infectious node recovers or one of the nodes becomes inactive, in the Markov process, the probability for transmission of infection through the link is $1-{e}^{-{p}_{0}\beta ({t}_{2}-{t}_{1})}$. But in our model this probability of transmission is ${p}_{0}(1-{e}^{-\beta ({t}_{2}-{t}_{1})})$ which is always smaller. Thus, we expect the infection probabilities in our model will be upper bounded by the probabilities from the Markov process which are in turn smaller than the values obtained from the N-intertwined approximation in equation 3. This property of equations 3 is particularly useful in controlling the infection spreading. In fact, if any initial infection that is governed by equation 3 dies out we know that the infection can not survive in our model.

### Model summary

Summarizing, the main definitions and assumptions of the network and the spreading model are the following:

- Nodes represent individuals, and links represent partnerships.
- Nodes can be in four states: inactive and susceptible, inactive and infected, active and susceptible, active and infected.
- The disease dynamics follows a Markovian susceptible-infected-susceptible (SIS) model to account for the possibility of recovery without immunity. Infection transmission rates and recovery rates are respectively *β* and *δ*.
- The sexual network has two layers; layer ${{\mathbb{L}}}_{1}$ representing partnerships that last several infectious periods of the disease under consideration –this layer can be sparse– and layer ${{\mathbb{L}}}_{2}$ with potential links that represents possible casual partnerships with duration comparable to the spreading dynamics –this layer is assumed to be connected.
- A potential link can transmit infection only if both individual at the ends of the link are active, thus we obtain partnerships with limited duration.
- Transitions of nodes between active and inactive states are independent Markovian processes with rates *γ*_1_ and *γ*_2_.

## Epidemic Threshold

In this section, we analyze the disease-free equilibrium of the SIS spreading equations 3, and we find a condition that guarantees the exponential die out of any small initial infection that is introduced in the population. A bifurcation analysis similar to the one in^10,42^ shows that, when this condition is not satisfied, there exists another equilibrium state that it is not disease-free. We first present the analysis when the network layers are random regular network, and later we provide the epidemic threshold for a generic network with an arbitrary structure.

Consider the case where ${{\mathbb{L}}}_{1}$ and ${{\mathbb{L}}}_{2}$ are regular random networks of degree *k*_1_ and *k*_2_, respectively. Moreover, let us assume that all the nodes have the same transition rates, i.e. ${\gamma }_{j}^{i}={\gamma }_{j} > 0\ \forall \ i$ (*j* = 1, 2). This means that, for any node, the probability of being active is *p*_2_ = *γ*_1_/(*γ*_1_ + *γ*_2_), and similarly for being inactive (*p*_1_ = *γ*_2_/(*γ*_1_ + *γ*_2_)). Hence, ${S}_{j}^{i}={p}_{j}-{I}_{j}^{i}$ (*j* = 1, 2). Introducing this relation in the previous system and summing the equations for the infected nodes in each state, we have

$$\begin{array}{lll}{\dot{I}}_{1} & = & (\beta {k}_{1}{p}_{1}-({\gamma }_{1}+\delta )){I}_{1}+(\beta {k}_{1}{p}_{1}+{\gamma }_{2}){I}_{2}\\  &  & -\beta {k}_{1}\sum _{j}\left(\sum _{i}{a}_{1}^{ij}{I}_{1}^{i}\right)({I}_{1}^{j}+{I}_{2}^{j})\\ {\dot{I}}_{2} & = & (\beta {k}_{1}{p}_{2}+{\gamma }_{1}){I}_{1}+\left(\beta {p}_{2}({k}_{1}+{p}_{0}{k}_{2})-({\gamma }_{2}+\delta )\right){I}_{2}\\  &  & -\beta \sum _{j}\left(\sum _{i}{a}_{1}^{ij}{I}_{2}^{i}\right)({I}_{1}^{j}+{I}_{2}^{j})-\beta {p}_{0}\sum _{j}\left(\sum _{i}{a}_{2}^{ij}{I}_{2}^{i}\right){I}_{2}^{j},\end{array}$$

where ${I}_{1}={\sum }_{i}{I}_{1}^{i}$ and ${I}_{2}={\sum }_{i}{I}_{2}^{i}$ are the expected number of inactive and active infected nodes, respectively. Let us now approximate the sums ${\sum }_{i}{a}_{l}^{ij}{I}_{l}^{i}$ by *k*_*l*_*I*_*l*_/*N*, which is a good approximation as long as the degree distribution has low variance (as in regular random or Ërdos-Rény networks) and the mean degree is high. Then, after dividing both sides of the equations by *N*, we have the following system of equations for the disease prevalence *ρ*_*j*_ = *I*_*j*_/*N* in each layer:

4 $$\begin{array}{lll}{\dot{\rho }}_{1} & = & (\beta {k}_{1}{p}_{1}-({\gamma }_{1}+\delta )){\rho }_{1}+(\beta {k}_{1}{p}_{1}+{\gamma }_{2}){\rho }_{2}\\  &  & -\beta {k}_{1}{\rho }_{1}({\rho }_{1}+{\rho }_{2})\end{array}$$

5 $$\begin{array}{lll}{\dot{\rho }}_{2} & = & (\beta {k}_{1}{p}_{2}+{\gamma }_{1}){\rho }_{1}+(\beta {p}_{2}({k}_{1}+{p}_{0}{k}_{2})-({\gamma }_{2}+\delta )){\rho }_{2}\\  &  & -\beta {\rho }_{2}({k}_{1}({\rho }_{1}+{\rho }_{2})+{p}_{0}{k}_{2}{\rho }_{2}).\end{array}$$

To study the linear stability of the disease-free equilibrium (DFE), we consider the Jacobian matrix of the previous system around the DFE

$${J}_{0}=\left(\begin{array}{ll}\beta {k}_{1}{p}_{1}-({\gamma }_{1}+\delta ) & \beta {k}_{1}{p}_{1}+{\gamma }_{2}\\ \beta {k}_{1}{p}_{2}+{\gamma }_{1} & \beta {p}_{2}({k}_{1}+{p}_{0}{k}_{2})-({\gamma }_{2}+\delta )\end{array}\right).$$

One can see that the discriminant Δ of the characteristic equation $\det ({J}_{0}-\lambda I)=0$ is always positive. Precisely, after some algebra and using that *p*_1_ + *p*_2_ = 1, we end up with

$$\Delta ={(\beta ({k}_{1}-{k}_{2}{p}_{0}{p}_{2})+{\gamma }_{1}+{\gamma }_{2})}^{2}+4\beta {k}_{2}{p}_{0}{p}_{2}({\gamma }_{1}+\beta {k}_{1}{p}_{2}) > 0,$$

which implies that *J*_0_ has two distinct real eigenvalues *λ*_1_ > *λ*_2_. Therefore, to guarantee that *λ*_1_ traverses 0 when using a tuning parameter of interest, we need that trace(*J*_0_) < 0. Then, the condition for *λ*_1_ = 0 follows from $\det ({J}_{0})=0$ which is equivalent to

6 $$\beta {k}_{2}{p}_{0}{p}_{2}(\beta {k}_{1}{p}_{1}-({\gamma }_{1}+\delta ))=(\beta {k}_{1}-\delta )({\gamma }_{1}+{\gamma }_{2}+\delta ).$$

**Figure 3:**
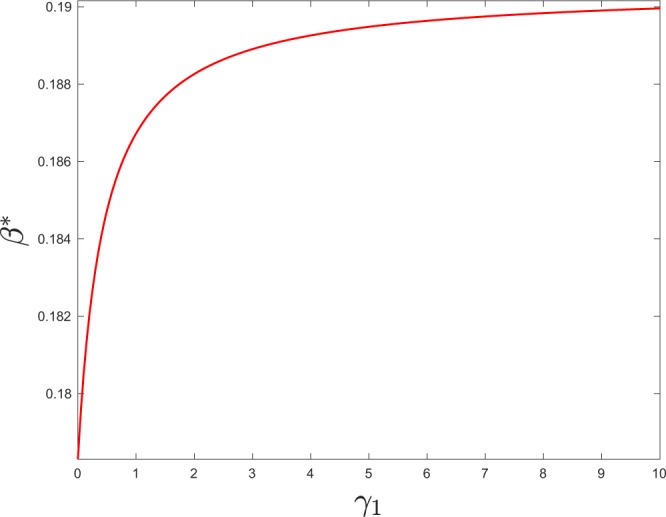
Critical value of *β* as a function of *γ*_1_ in regular random networks. Parameters: *k*_1_ = 4, *k*_2_ = 50, *p*_0_ = 0.1, *δ* = 1, *γ*_2_ = *γ*_1_ (*p*_2_ = 0.5).

The previous condition defines a polynomial of degree 2 for the critical value of *β*, β^*^. It is easy to see that this equation has two real roots 0 < *β*_1_ < *β*_2_. Since we want the value of *β* for which *λ*_1_ goes from negative to positive, β^*^ = *β*_1_. Fig. 3 shows the dependence of β^*^ with the transition rate *γ*_1_ obtained by solving the previous equation for *γ*_1_ = *γ*_2_. So, in this figure, the probability *p*_2_ for a node of being active is always 1/2. However, although a node always spends half of its time with partners in ${{\mathbb{L}}}_{2}$, the figure reveals that how it visits this layer (short and frequent visits or longer but less frequent ones) affects the spread of the disease.

**Figure 4:**
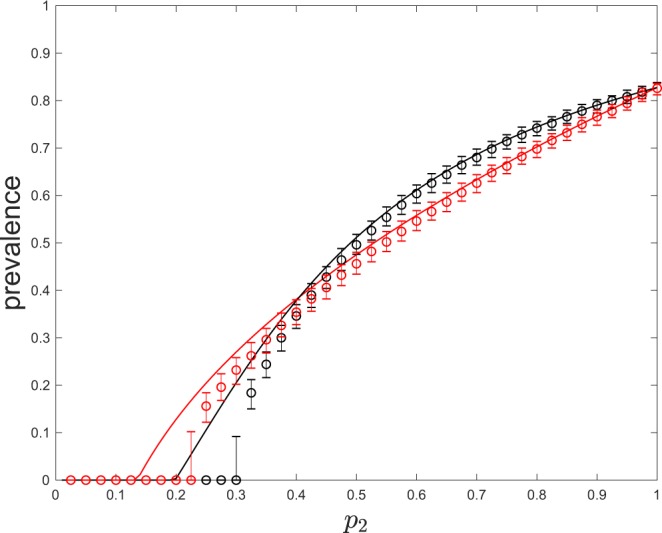
Disease prevalence as a function of *p*_2_ in regular random networks. Circles show, for each set of parameters values, the median of the prevalence in networks of size 500 after 1000 runs of the Markov process approximated by the mean-field model, and error bars show the corresponding interquartile range. Parameters: *k*_1_ = 4, *k*_2_ = 50, *p*_0_ = 0.5, *β* = 0.2, *δ* = 1, *γ*_1_ = 0.01 (red), *γ*_1_ = 10 (black).

A second feature of the mean-field (MF) model is the possibility of having a lower prevalence at the endemic equilibrium for values of *γ*_1_ leading to lower epidemic thresholds. We illustrate this fact in Fig. 4 where a bifurcation curve from the DFE is shown using the probability of being active, *p*_2_, as a tuning parameter. Note that, in order to have *p*_2_ as a bifurcation parameter, the epidemic has to die out if inactive individuals alone (*γ*_1_ = 0, *p*_1_ = 1) are not enough to sustain the epidemic, i.e., if *βk*_1_/*δ* < 1. As expected, the higher *p*_2_ is, the higher the prevalence because infection transmission routes in ${{\mathbb{L}}}_{2}$ are used longer. The figure also shows a quite surprising fact: although *γ*_1_ = 0.01 leads to a lower epidemic threshold in terms of *p*_2_ when compared to that of *γ*_1_ = 10, it also leads to a lower equilibrium prevalence for *p*_2_ > 0.37. We can also observe this feature of the solutions in the output of the simulations over regular random networks of the Markov process corresponding to the mean-field model (see section 2.2). These simulations have been performed using the Gillespie algorithm until a final time T = 600. Finally, Fig. 4 also reveals that the MF model underestimates the epidemic threshold observed from the stochastic simulations. As discussed in section 2.2, this is due to the higher infection probabilities assumed under the mean-field approach.

The analyze of disease-free equilibrium we presented for random regular networks can be generalized for any generic network structure with heterogeneous activity parameters. Indeed, we performed such an analysis and we have presented the details in the supplementary material, section 2. We have shown that there exists a threshold β^*^ such that for any value of transmission rate *β* < β^*^, the disease-free equilibrium in equation 3 is exponentially stable, i.e. any infection dies out. Value of the epidemic threshold β^*^ is

7 $${\beta }^{* }=\delta {\lambda }_{max}^{-1}({B}^{* }),$$

where ${\lambda }_{max}^{-1}({B}^{* })$ is the largest eigenvalue of

8 $${B}^{\star }=\left(\begin{array}{cc}{A}_{1} & {p}_{0}{p}_{2}{A}_{2}\\ {p}_{2}{A}_{1} & {p}_{0}{p}_{2}^{\star }{A}_{2}\end{array}\right),$$

and ${p}_{2}^{\star }$ is a diagonal matrix such that

$$\begin{array}{lll}{({p}_{2}^{\star })}_{i,i} & = & {p}_{2}^{i}\frac{1-{p}_{2}^{i}+{\bar{\gamma }}_{2}^{i}\,{p}_{2}^{i}}{1-{p}_{2}^{i}+{\bar{\gamma }}_{2}^{i}},{\bar{\gamma }}_{2}^{i}={\gamma }_{2}^{i}/\delta ,{p}_{2}^{i}=\frac{{\gamma }_{1}^{i}}{{\gamma }_{2}^{i}+{\gamma }_{1}^{i}}.\end{array}$$

We can see this threshold depends not only on *p*_2_ and *p*_0_ but also on *γ*_2_. Hence, it captures the effect of the probability for the existence of a casual link and its duration. Indeed, in section 2.1 we explained that the probability for the existence of a temporal link between any nodes *i*, *j* (casual partnership) in the steady state is ${p}_{2}^{i}{p}_{2}^{j}{p}_{0}$ and the expected duration of the link is ${({\gamma }_{2}^{i}+{\gamma }_{2}^{j})}^{-1}$. Thus, this threshold value is different from that of an static network with a link of weight ${p}_{2}^{i}{p}_{2}^{j}{p}_{0}$ between nodes *i* and *j*.

One application of equation 7 is in control of spreading processes. For example, in Fig. 4 of the supplementary material, we have shown the prevalence of the infection obtained from the simulations of the exact process (depicted in Fig. 2b) as a function of time . Since in those simulations we kept the transmission rates *β* below the threshold value, the infection prevalence dies out.

## Simulations

In the following, we present the results from the simulation of the exact process, which we described in section 2.2, over random regular networks. Similar simulations over a heterogeneous network structure are included in section 3 of the supplementary material. These simulations clarify the relation between the exact process and its approximating counterpart, which are the N-intertwined equations and the stochastic Markov process (Fig. 2a) we described in section 2.2. In addition, using the simulations we explore effect of the model parameters on the infection spreading. The link to the public repository for the simulation code is provided in the Data Availability section.

As usual in the setting of continuous-time stochastic simulations, we use the well-known Gillespie algorithm^43^ in all experiments. All random regular networks are generated using the configuration model algorithm^44^. We run this algorithm twice and independently to get layers ${{\mathbb{L}}}_{1}$ and ${{\mathbb{L}}}_{2}^{}$. To get the empty intersection condition, we extract ${{\mathbb{L}}}_{2}$ from ${{\mathbb{L}}}_{2}^{{\prime} }$ by deleting the links that are common to ${{\mathbb{L}}}_{1}$. When the respective prescribed degrees *k*_1_ and *k*_2_ are small with respect to the number *N* of nodes, this happens with very small probability, and the obtained graph ${{\mathbb{L}}}_{2}$ has a mean degree very close to *k*_2_. The reported experimental prevalence values are always computed by averaging over several hundreds of independent realizations of the stochastic process, each corresponding to a particular random initial condition with a fixed number of infected individuals (see the captions of figures for more details on each experiment). Since the average prevalence does not show the distribution of prevalence across independent simulations, we calculated the median and the interquartile range and show them as error bars in the figures.

**Figure 5:**
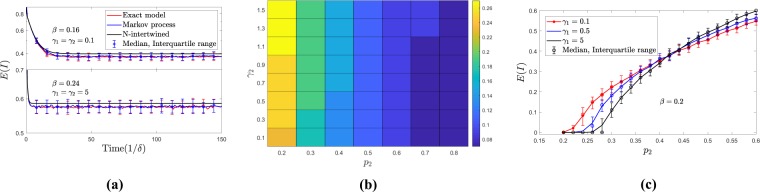
Results of numerical and stochastic simulations of the spreading processes on random regular graphs, discussed in section 4. Panel (**a**) shows the comparison of different approximate processes with the exact process; panel (**b**) shows the epidemic threshold of the exact process, as a function of *p*_2_ (probability of being active in ${{\mathbb{L}}}_{2}$) and the parameter *γ*_2_, which is proportional to the inverse of expected duration of active potential links; panel (**c**) shows how the infection prevalence in the metastable state is affected by different parameters in the exact process. Error bars show the median and the interquartile range.

Here we assumed a population of 500 nodes, and for the layer ${{\mathbb{L}}}_{1}$, we generated a random regular network where each node has four neighbors, while for the layer ${{\mathbb{L}}}_{2}$ we used a random regular network of degree 50. Moreover, for the layer ${{\mathbb{L}}}_{2}$ we have assumed *p*_0_ = 0.5. In these simulations, to estimate the expected number of infected nodes in the Markov process and the exact process, we generated 200 independent realizations and calculated the average, median and interquartile range for the total number of infected nodes over these realizations at different times. Moreover, we assumed that all the nodes were infected and active at *t* = 0. Figure 5a shows the infection prevalence curves obtained from the N-intertwined approximation, the Markov process, and the exact spreading process. As we expect, the N-intertwined equations provide an upper bound for the prevalence values obtained from the Markov process and the exact process.

### Impact of partnership duration on the epidemic

In Fig. 5b, we have shown the epidemic threshold, obtained from the simulation of the exact process, as a function of probability of being active, *p*_2_, and *γ*_2_, which is proportional to the inverse of expected duration of active links. From this figure, we can see that, when *p*_2_ increases, the epidemic threshold decreases. However, when the number of active nodes is small (lower values of *p*_2_), the epidemic threshold increases as the duration of links decreases (larger values of *γ*_2_). This effect of the link duration in ${{\mathbb{L}}}_{2}$ on the epidemic threshold is also clear from Fig. 5c. In this figure, we see that reaching the metastable state requires less active nodes (smaller *p*_2_) when the link duration is longer (note that, for a given *p*_2_, *γ*_2_ is proportional to *γ*_1_).

Figure 5c,b show that the epidemic threshold decreases with increasing link duration and this result can appear counter-intuitive, especially when we consider sexual networks. For instance, one can conclude that, due to the higher epidemic threshold, a population in which the sexual behavior is dominated by short-duration casual partnerships is less vulnerable to epidemics, compared to a population with longer-duration casual partnerships. We can interpret this result thinking about two counterbalancing processes; the probability of transmission during the partnership duration, and the frequency of changing partners. With short-duration casual partnerships, the number of partners in a given interval is greater but the probability of transmission is smaller; with long-duration casual partnerships, the number of partners is smaller but the probability of disease transmission is higher. Our numerical simulations show that these two processes do not obtain a complete balance; rather, the duration of the partnership plays a more important role than the number of partners. For this reason, the threshold is increased by short-duration partnerships in spite of the increased number of partners.These results are obtained keeping a constant value for the infection transmission rate.

### Partnership duration and number of sexual intercourse

To consider a different but also important viewpoint, we can compare the two scenarios (long-duration casual partnership and short-duration casual partnership) keeping the same probability of infection transmission per sexual intercourse, instead of the same infection probability per unit of time. In this case, we impose a similar average number of sexual intercourse in these two scenarios, which in turn corresponds to different infection rates of the disease. To understand the difference, assume a population where the average number of sexual intercourse for an individual in a long-term casual partnership is once per week, and the probability of infection transmission in an intercourse with an infected individual is 0.5. Consequently, the infection transmission rate in partnerships with duration significantly larger than a week can be estimated as ${\beta }_{p}=-{\rm{ln}}\,(0.5)/7=0.1{{\rm{day}}}^{-1}$. Indeed, we have set the value of *β*_*p*_ such that, if a partner is infected, the probability that the susceptible partner stays susceptible is multiplied by one half each week during an infection period of length *T* days; in other words, ${e}^{-{\beta }_{p}T}={(0.5)}^{T/7}$. In contrast, assuming the same probability of infection during an intercourse with an infected individual, for short-duration partnerships with one intercourse, we can assign the transmission rate by solving

9 $${\int }_{0}^{\infty }{\alpha }^{-1}{e}^{-t/\alpha }(1-{e}^{-\beta t})dt=0.5,$$

for *β*, while for consistency in the mathematical modeling, we can assume a duration of *α* = 0.5 day for short-duration partnerships. In the equation above, *α*^−1^*e*^−*t*∕*α*^ is the probability density for a link with duration *t*, and 1 − *e*^−*βt*^ is the probability for transmission of infection from an infected node within the period that the link exists. Thus, the integral in equation 9 gives the expected value of transmission probability, which is easily computed. A simple expression for *β* follows from equation 9, namely, *β* = *α*^−1^. Therefore, the equivalent transmission rate for a short-duration partnership becomes *β*_*e*_ = 2 day^−1^.

**Figure 6:**
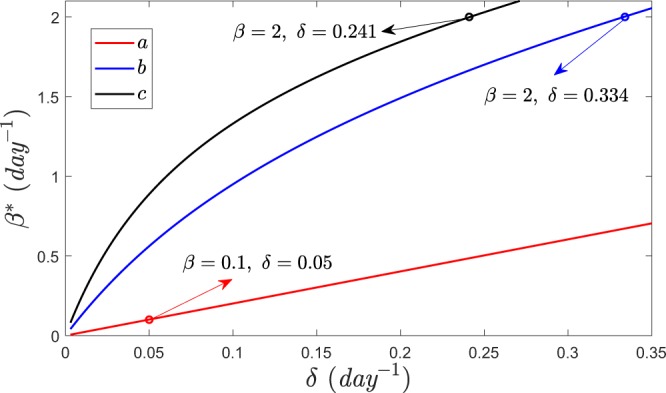
Infection transmission rate threshold as a function of recovery rate for three different temporal networks discussed in section 4. Case **a** corresponds to partnerships of 60 days duration and cases **b,c** correspond to casual sexual encounters.

In order to compare the vulnerability of populations with different duration of partnership under this viewpoint, in Fig. 6 we have plotted the epidemic threshold, obtained from equation 6, corresponding to three different sets of model parameters,

- $${\gamma }_{2}^{-1}=120$$ days, $${\gamma }_{1}^{-1}=1$$ day, *l* = 1, *k*_1_ = 0
- $${\gamma }_{2}^{-1}=1$$ day, $${\gamma }_{1}^{-1}=6$$ days, *l* = 1, *k*_1_ = 0
- $${\gamma }_{2}^{-1}=1$$ day, $${\gamma }_{1}^{-1}=13$$ days, *l* = 1, *k*_1_ = 0

Among these selections of parameters, case **a** corresponds to partnerships with an average duration of ${(2{\gamma }_{2})}^{-1}=60$ days. Moreover, using equation 2, we set *k*_2_*p*_0_ such that the average number of links in each activity period is *l* = 1. For simplicity, we assumed that there is no static links, *k*_1_ = 0. Contrarily, cases **b** and **c** correspond to sexual encounters with frequencies that are once per week or two weeks, respectively. Indeed, case **c** is more comparable to case **a** because they provide a similar average number of sexual intercourses per year.

From the curve for case **a**, in Fig. 6  we conclude that the epidemic threshold β^*^ is greater than the estimated value of transmission rate in partnership, *β*_*p*_ = 0.1, when the average recovery rate, *δ*, is greater that 0.05day^−1^ or, equivalently, for the expected average recovery time of *δ*^−1^ < 20 days. Hence, the infection dies out when the recovery time is smaller than 20 days. On the other hand, for case **c**, which corresponds to sexual encounters with frequency of once per two weeks, the transmission rate *β*_*e*_ = 2 is smaller than the epidemic threshold only for the expected recovery time *δ*^−1^ < (0.241)^−1^ ≊ 4 days. This suggests that the population with sexual encounter behavior is more vulnerable than the population with partnership behavior.

## Conclusions

In this paper, we have developed a network model that incorporates the process of switching between two network layers –steady and casual partners– driven by individual activities, which define the propensity of individuals to be engaged in casual partnerships. Hence, the temporal characteristic of the model appears as a consequence of the existence of such partnerships. This scenario is suitable for studying the dynamics of sexually transmitted diseases in real communities where casual partners are not always disclosed in partner-notification programs. These partnerships are modeled by considering the activation of links drawn from a set of potential links. Each of these links is activated with probability *p*_0_ when the nodes at both ends are willing to develop a casual partnership, i.e., when both nodes are active.

The model incorporates two ingredients, namely, change of partners and partnership duration, that have also been discussed in pair formation models. The contribution of casual partnerships to the disease spread by increasing the basic reproduction number has already been highlighted in these models (see, for instance, Eq. (5) in^6^ and Eq. (3) in^41^). Here, our model allows to assess the role of the layer of casual partnerships by quantifying its utilization through the activity probability parameter *p*_2_, and by considering the mean duration of partnerships by means of *γ*_2_. In particular, we have studied how different parameters of the model affect the epidemic threshold and the disease prevalence in the metastable state –endemic equilibrium of the mean-field model. We have found that, given a fixed value of the infection transmission rate *β*, the prevalence of infection strongly depends on the utilization of the layer of casual partnerships and on the duration of these partnerships. Our simulations show that the epidemic threshold decreases with increasing link duration, while short partnership durations decrease the probability of disease transmission, thus increasing the threshold. Finally, and without contradiction, our analysis shows that casual sexual behavior, which implies extremely short partnerships, makes the population vulnerable to the infection spreading.

## Supplementary information

Supplementary Information.

## Data Availability

The simulation code for this study is available from https://github.com/KSUNetSE/Timvar. The authors are willing to provide further details upon request.
