# Supplementary material for: A multilayer temporal network model for STD spreading accounting for permanent and casual partners
Source: Sci Rep. 2020 Mar 2;10:3846. doi: 10.1038/s41598-020-60790-0 (PMC7052224; doi:10.1038/s41598-020-60790-0)
Supplement: Supplementary file 1 — Supplementary Information. [file 41598_2020_60790_MOESM1_ESM.pdf]

Supplementary Information

A multilayer temporal network model for STD spreading accounting for permanent and casual partners

Aram Vajdi, David Juher, Joan Saldaña, Caterina Scoglio

Contents

1 Temporal characteristics of the network structure **1**

2 The disease-free equilibrium on general networks **2**

3 Simulations on a real-world network structure **4**

References **6**

1 Temporal characteristics of the network structure

In the section 2.1 of the main manuscript we have described the two layer temporal network model. Here, we calculate the probability that any two nodes i, j develop a link, during the period that node i is active.

Based on the description of the model, when node i becomes active, node j is active with probability p_2^j and they develop a link with probability p_0 or the link formation fails with probability the $1-p_0$. Assuming they do not develop link, node j has another possibility to develop link with i, if it becomes inactive and active again before node i becomes inactive. Assuming that the two nodes are active, the probability that node j becomes inactive sooner than node i is $f_1 = \gamma_2^j/(\gamma_2^j + \gamma_2^i)$. This stems from the fact that for any two competing exponential processes A and B, the probability that A will be the minimum is $rate_A/(rate_B + rate_A)$. Now, if we assume that node j became inactive sooner than i, the probability that node j becomes active again before node i goes to the inactive state is $f_2 = \gamma_1^j/(\gamma_1^j + \gamma_2^i)$. In summary, the probability that the two nodes develop a link in a second trial, assuming the first trial fails, is $f_1 f_2 p_0$.

Figure 1 depicts the process we described above. Moreover, in that figure we have accounted for the possibility that the node j might be initially inactive when node i becomes active. Hence, accounting for all the possibilities shown in figure 1, and allowing for higher number of link development trials, we can obtain the probability for the establishment of a link between nodes i and j while node i is active as

$$P_{j|i} = p_2^j p_0 \sum_{r=0}^{\infty} (f_1 f_2 (1-p_0))^r + (1-p_2^j) f_2 p_0 \sum_{r=0}^{\infty} (f_1 f_2 (1-p_0))^r = \frac{p_2^j p_0}{1 - f_1 f_2 (1-p_0)} \left(1 + \frac{p_1^j}{p_2^j} f_2\right), \tag{1}$$

where $p_1^j = \gamma_2^j/(\gamma_2^j + \gamma_1^j)$ and $p_1^j + p_2^j = 1$. To confirm this result we performed a simulation to find the probability of existence of link between the nodes i, j during the periods that i is active and the probability we obtained from the simulation perfectly matches this theoretical result.

If we assume that node i has k_2 neighbors in the layer $\mathbb{L}_2$ and all these nodes have the same activity rates, γ_1, γ_2, then the distribution of number of developed links, during the period that i is active, follows binomial distribution $P(N_L = r) = \binom{k_2}{r} p_L^r (1-p_L)^{k_2 - r}$ where $p_L = P_{j|i}$ is obtained from equation 1. Moreover, duration of the links is exponentially distributed with the rate $2\gamma_2$. Figure 2a shows the probability mass function of number of developed links obtained from a simulation in which we counted number of established links in each period that node i was active. For this simulation we assumed that node i has $k_2 = 100$ potential neighbors in the layer $\mathbb{L}_2$. In addition, figure 2b shows the distribution of links durations. These figures show the result of simulation follows the expected theoretical distributions.

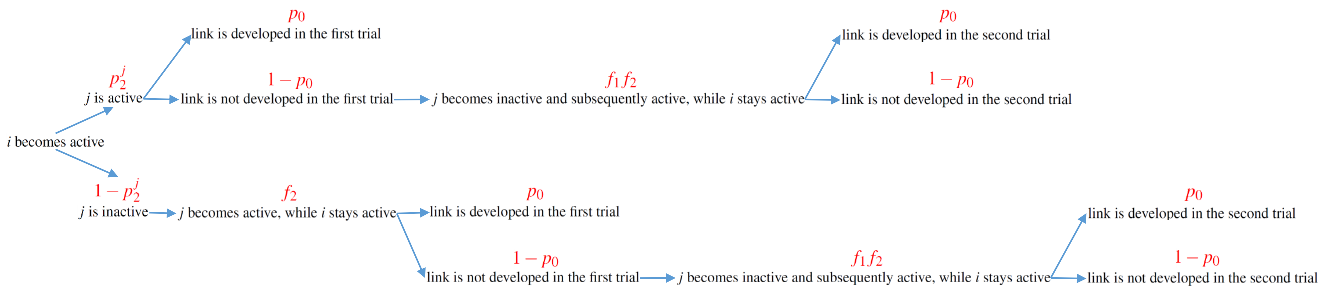

Figure 1. Different processes that result in link establishment between nodes i, j while node i is active. The values in red are the probabilities for each step in the process.

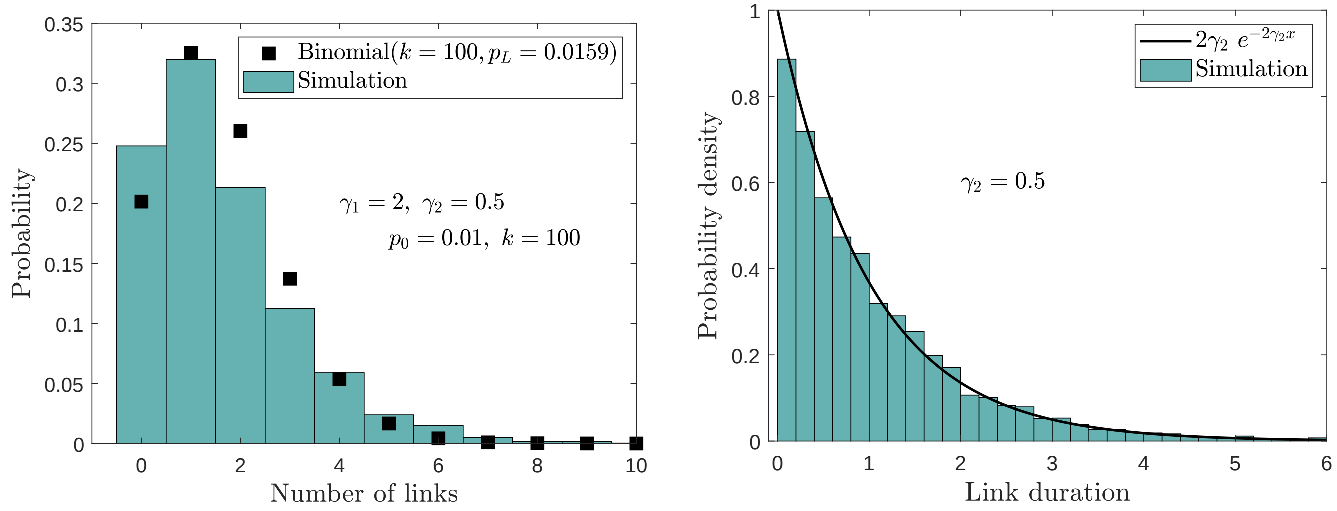

(a) **(b)**

Figure 2. Distribution of number of developed links (**a**) and their duration (**b**), during the period a node is active. The relevant model parameters are shown in panel (**a**).

2 The disease-free equilibrium on general networks

In this section we focus on the stability analysis of the disease-free equilibrium of the SIS spreading equations (equation (3) in the main manuscript), and we find a condition that guarantees the exponential die out of any small initial infection that is introduced in the population.

For the SIS spreading equations, it is a straightforward observation that the disease-free state given by

$$S_1^i = p_1^i = \frac{\gamma_2^i}{\gamma_2^i + \gamma_1^i},\ \ S_2^i = p_2^i = \frac{\gamma_1^i}{\gamma_2^i + \gamma_1^i},\ \ I_1^i = 0,\ \ I_2^i = 0, \tag{2}$$

is an equilibrium state. In equation (2), p_1^i and p_2^i are the probabilities that node i is active and inactive, respectively, at the steady-state of the continuous-time Markov chain that governs the activity of node i. Here, we study the evolution of the initial infection around the disease-free equilibrium using the corresponding linearized version of SIS spreading equations. In the analysis that comes later, we use state variables $I^i = I_1^i + I_2^i$ and I_2^i instead of I_1^i, I_2^i. Particularly, this choice of variables directly leads to a relation between the network structure and the model parameters such that, if it is satisfied, the disease-free equilibrium is exponentially stable. If we choose I_1^i, I_2^i, we would need extra algebraic manipulation to get the same relation.

If $\mathbf{I}^i$ and $\mathbf{I}_2^i$ represent small perturbations from the disease-free equilibrium, using the linearized version SIS spreading

equations we obtain the following linear dynamical system

$$\dot{\mathbf{I}}^i = -\delta \mathbf{I}^i + \beta \sum_j a_1^{ij} \mathbf{I}^j + \beta' \sum_j a_2^{ij} p_2^i \mathbf{I}_2^j, \tag{3a}$$

$$\dot{\mathbf{I}}_2^i = -(\gamma_2^i + \gamma_1^i)\mathbf{I}_2^i + \gamma_1^i \mathbf{I}^i - \delta \mathbf{I}_2^i + \beta \sum_j a_1^{ij} p_2^i \mathbf{I}^j + \beta' \sum_j a_2^{ij} p_2^i \mathbf{I}_2^j, \tag{3b}$$

that determines the evolution of the state variables

$$X = (\mathbf{I}^1, \cdots, \mathbf{I}^N, \mathbf{I}_2^1, \cdots, \mathbf{I}_2^N).$$

We can write equations (3) as $\dot{X} = JX$ where $J = B - D$ with

$$B = \begin{pmatrix} \beta A_1 & \beta' p_2 A_2 \\ \beta p_2 A_1 + \gamma_1 & \beta' p_2 A_2 \end{pmatrix}, \quad D = \begin{pmatrix} \overline{\delta} & 0 \\ 0 & \overline{\delta} + \gamma_1 + \gamma_2 \end{pmatrix}.$$

In the definition of matrices B and D above, $p_2, \gamma_1, \gamma_2, \overline{\delta}$, are diagonal matrices whose entries are the corresponding parameters for different nodes. It is well known that the linear system is stable if the stability modulus

$$\alpha(J) := \max\{\Re(\lambda) | \lambda \in \text{spectrum of } J\} < 0.$$

In the following we show there exists a threshold β^* such that for any value of transmission rate $\beta < \beta^*$ the disease-free equilibrium is exponentially stable, i.e. $\alpha(J) < 0$.

Lemma 1. *If $\gamma_1 > 0$, $p_0 > 0$ and $\beta > 0$, then the following statements hold:*

a) There is a real eigenvalue of J, denoted by $\lambda_{\max}(J)$, such that any other eigenvalue λ satisfies $\Re(\lambda) \leq \lambda_{\max}(J)$, and the eigenvector Z corresponding to $\lambda_{\max}(J)$ is unique and positive, $Z > 0$.

b) $\min_i \sum_k J_{ik} \leq \lambda_{\max}(J) \leq \max_i \sum_k J_{ik}$

c) If there exists a vector $X \geq 0$ such that $JX \leq \mu X$, then $X > 0$ and $\lambda_{\max}(J) \leq \mu$ with $\lambda_{\max}(J) = \mu$ if and only if X is a multiple of Z.

Proof. Let us prove that the matrix B is irreducible. Consider the associated graph G_B with $2N$ nodes $\{v_1, \ldots, v_N, w_1, \ldots, w_N\}$ such that there is a directed link from node i to node j if and only if $B_{ij} > 0$. As it is well known, B will be irreducible if and only if G_B is strongly connected, that is, for any pair of nodes x, y there is a path of links in G_B from x to y. Recall that, by hypothesis, $\mathbb{L}_2$ is connected and, in consequence (since A_2 is symmetric), strongly connected. Observe that $p_2 > 0$, because $\gamma_1 > 0$. The following facts about the structure of the graph G_B follow from the four blocks defining the matrix B:

A) The block βA_1 implies that the subgraph of G_B induced by the nodes $\{v_1, \ldots, v_N\}$ is isomorphic to $\mathbb{L}_1$

B) The lower right block $\beta' p_2 A_2$ implies that the subgraph of G_B induced by the nodes $\{w_1, \ldots, w_N\}$ is isomorphic to $\mathbb{L}_2$

C) The diagonal entry γ_1 in the block $\beta p_2 A_1 + \gamma_1$ implies that there is a link from w_i to v_i for every $1 \leq i \leq N$

D) The upper right block $\beta' p_2 A_2$ implies that for every $1 \leq k \leq N$ there are links from v_i to some nodes w_k.

Now, to prove that there is a path between any pair of nodes in G_B, we must consider four cases. If the pair has the form $\{v_i, v_j\}$, by (D) there is a link from v_i to some w_k, by (B) there is a path from w_k to w_j (since $\mathbb{L}_2$ is strongly connected) and by (C) there is a link from w_j to v_j. The existence of a path for the three remaining forms of the pair, $\{v_i, w_j\}$, $\{w_i, v_j\}$ and $\{w_i, w_j\}$, follows analogously using (A–D).

From the definition of J, we have $J = B - D$ where B is a non-negative matrix and D is a nonnegative diagonal matrix. If we assume $\tau = \max_k D_{kk}$ then matrix $C = B - D + \tau I$, with I denoting the identity matrix, is also nonnegative. Since B is irreducible, then C becomes irreducible. Now we can use Perron-Frobenius theorem for non-negative irreducible matrices[1] to show the statements of Lemma 1 hold for the matrix $C = J + \tau I$. Since the eigenvectors of J are similar to the eigenvectors of C and the eigenvalues of J can be obtained by subtracting τ from the eigenvalues of C, we deduce the statements of Lemma 1 also hold for J. □

If we assume β^* is the transmission rate for which $\lambda_{\max}(J_{\beta^*}) = 0$ and $Z_{\beta^*} > 0$ is the corresponding eigenvector, using Lemma 1 it is straightforward to show that for any $\beta < \beta^*$ we have $J_\beta Z_{\beta^*} \leq 0$. Next, we can use the last part of Lemma 1 and conclude $\lambda_{\max}(J_\beta) < 0$. This shows that, if $\beta < \beta^*$, the disease-free equilibrium is exponentially stable. Moreover, to prove the existence of β^*, we can use statement (b) of Lemma 1 and consider the limiting cases $\beta \to 0$ and $\beta \to \infty$ to show that there are β_1 and β_2 such that $\lambda_{\max}(J_{\beta_1}) < 0$ and $\lambda_{\max}(J_{\beta_2}) > 0$. Since $\lambda_{\max}(J_\beta)$ is a continuous function of β there should be a β^* such that $\lambda_{\max}(J_{\beta^*}) = 0$.

In the proof of Lemma 1, the irreducibility condition on B was derived from the positivity of the rates γ_1, p_0 and β and our standing assumption that both layers were connected. If for some reason one wants to relax these assumptions, then one cannot assure that the graph G_B of the proof is strongly connected. In this case, we can separate it into strongly connected components and the threshold analysis which was presented in this section can be done on different components separately. Particularly, for an individual i that never gets active we have $\gamma_1^i = 0$ or equivalently $p_2^i = 0$. In such a case we can see the node that corresponds to I_2^i in the associated graph G_B is disconnected from the rest of nodes and the threshold analysis can be carried out by eliminating the row and column for I_2^i in the J matrix. In fact, if in the matrix J we exclude all those rows and columns that correspond to I_2 for the individuals that never get active we can see the resulting matrix B is irreducible if and only if union of the two layers, $\mathbb{L}_1$ and $\mathbb{L}_2$, is strongly connected.

As we have shown, the threshold value β^* is the smallest transmission rate β for which the eigenvalue problem $J_\beta Z = 0$ has a nontrivial solution. Writing $Z = (Z_1, Z_2)^T$ with $Z_1 = \mathbf{I}$ and $Z_2 = \mathbf{I}_2$, we have $Z_1 = \beta/\delta\,(A_1 Z_1 + p_0 p_2 A_2 Z_2)$ from equaling the r.h.s. of (3a) to 0. Then, replacing Z_1 by this expression in the term $\gamma_1^i Z_1^i$ of the r.h.s. of (3b) and rearranging terms, we can rewrite the eigenvalue problem as $\tau B^\star Z = Z$, where

$$B^\star = \begin{pmatrix} A_1 & p_0 p_2 A_2 \\ p_2 A_1 & p_0 p_2^\star A_2 \end{pmatrix}, \tag{4}$$

$\tau = \beta/\delta$ is the so-called effective spreading rate[2], and $p_2^\star$ is a diagonal matrix such that

$$(p_2^\star)_{i,i} = p_2^i \frac{1 - p_2^i + \overline{\gamma}_2^i p_2^i}{1 - p_2^i + \overline{\gamma}_2^i}$$

with $\overline{\gamma}_2^i = \gamma_2^i/\delta$. From the expression $\tau B^\star Z = Z$, we can find the threshold value β^* from

$$\tau^* = \frac{\beta^*}{\delta} = \lambda_{max}^{-1}(B^*). \tag{5}$$

3 Simulations on a real-world network structure

In this section, we use the largest connected component of a network that represents sexual contacts among men who have sex with men in the city of San Francisco[3]. This network has 953 nodes and 1011 links, where a few nodes with high degrees act as hubs. Although some of the links in this network are temporal, for this experiment, we treated all these links as permanent and defined the network layer $\mathbb{L}_1$ from them. Next, we generated $\mathbb{L}_2$ as a synthetic network using the distance between the nodes in $\mathbb{L}_1$. It is possible to define different types of closeness for any two nodes in a connected graph like, for example, the shortest path distance or the resistance distance. Here we used the resistance distance and calculated the closeness of any two nodes in $\mathbb{L}_1$. To generate the neighborhood set in $\mathbb{L}_2$ of any node v, we assumed that all nodes with a distance to v smaller than a threshold value are the neighbors of v, excluding those nodes that already have a permanent link with v in $\mathbb{L}_1$. Moreover, for the layer $\mathbb{L}_2$ we have assumed $p_0 = 0.5$. Although for this experiment we generated $\mathbb{L}_2$ using the closeness in the layer $\mathbb{L}_1$, in real-world applications we need to consider other types of relations between the nodes in the process of inferring potential partners. One of these relations can be, for instance, the geographical distance.

In Fig. 3a, we compared the prevalence of infection obtained from three different processes discussed in section 2.2 of the main manuscript. We define prevalence as the average of nodal infection probabilities. To estimate the prevalence at different points in time, we calculated the average of the infected population over 500 simulations of the processes. As initial condition, we have assumed that all the nodes are active and infected at $t = 0$. In this figure, the curves referred to as "N-intertwined" show the prevalence calculated from the solution of equation (3) in the main manuscript. In the same figure, the curves that are labeled as "Markov process", are calculated using stochastic simulations. In this auxiliary process a potential contact in $\mathbb{L}_2$ transmits infection with probability βp_0 whenever the nodes at both ends of the link are active. Finally, in Fig. 3a we have also included the prevalence calculated using the stochastic simulations of the exact spreading model where the active nodes develop a contact over the potential links with probability $p_0 = 0.5$. The results of these simulations are the curves tagged as "Exact model". Based on our discussion in section 2.2 of the main manuscript, we expect that the prevalence obtained from the "N-intertwined" equations will be higher than that of the "Markov process" at any time. Moreover, we also expect that the

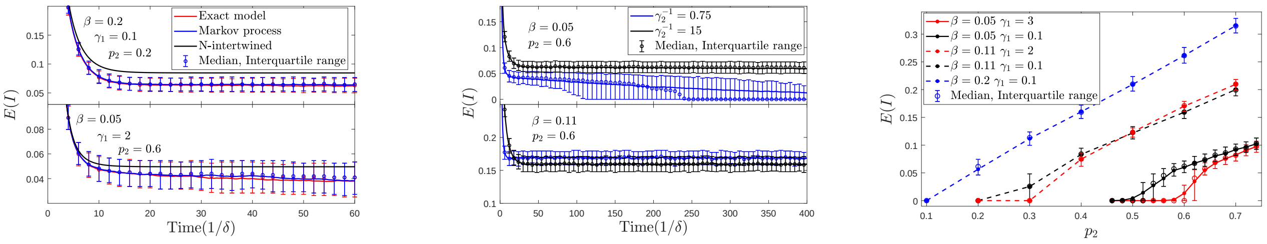

(a) **(b)** **(c)**

Figure 3. Results of the numerical and stochastic simulations discussed in section 3. For the Markov process and the exact process curves, we generated 500 independent realizations of these stochastic processes and calculated the average, median and interquantile range for number of infected nodes across these realizations
. In these simulations, all the nodes were infected and active at $t = 0$. Panel (a) compares the prevalence according to the two approximate processes and the exact spreading process; panel (b) shows the effect of link duration on the epidemic threshold in the exact process; panel (c) shows how the epidemic threshold is affected by different parameters in the exact process.

prevalence in the "Markov process" will be an upper bound for the "exact model". We clearly see such a relation between the prevalence curves in Fig. 3a. In fact, we repeated the simulations with different sets of parameters values and we observed the same trend.

In another experiment, we studied the effect of temporal links' duration on the prevalence of infection, when the exact spreading model is unfolding over the network. Figure 3b shows the curves obtained from the simulations. From this figure we can observe that, for $\beta = 0.05$, the spreading process with $\gamma_2^{-1} = 15$ reaches metastability, while with $\gamma_2^{-1} = 0.75$ infection dies out. Since for this experiment we kept $p_2 = 0.6$ and $p_0 = 0.5$, in the steady state the probabilities for the existence of a temporal link between any two nodes are similar across different curves in Fig. 3b. In other words the average number of casual partners for any node is the same for the two different values of γ_2. Hence, the only difference between the curves with different activity rates is the duration of the links, which has an expected value of $(2\gamma_2)^{-1}$. In the simulations we observe that, when the duration of temporal links are longer, $\gamma_2^{-1} = 15$, the epidemic threshold value is indeed smaller.

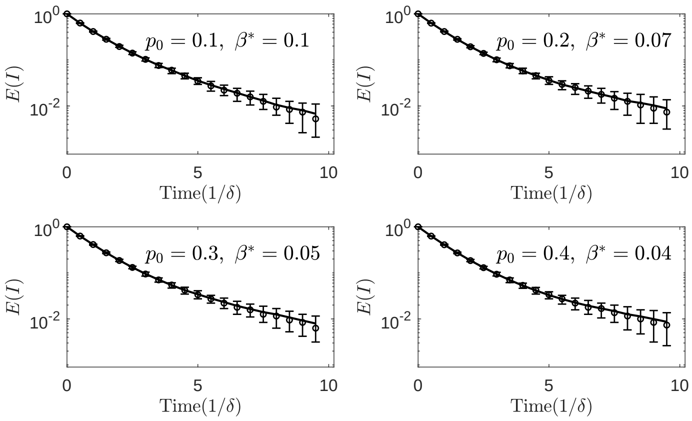

Figure 4. This figure shows that the approximate threshold $\beta^\star$ obtained from the N-intertwined equations is a lower-bound for the exact epidemic threshold. Different plots show the prevalence of infection in the simulation of the exact process for different configurations in the parameters space. In this experiment, for each node, we assigned random values, between zero and three, to the corresponding rates γ_1 and γ_2. In all the simulations we have assumed β is slightly lower than $\beta^\star$. Since, $\beta^\star$ provides a lower-bound for the exact epidemic threshold, we can see the infection dies out in all the simulations. The curves show average number of infected nodes over 400 realizations of the process. The median and the interquartile range for some times are also shown (error bars).

Although Fig. 3b shows that link duration can change the course of spreading process, more simulations reveal that when nodes are active with high probabilities, link duration is not as effective in the infection spreading as in the case where these nodal probabilities are low. When nodal probabilities of being active are high, if a node becomes active, then there is a high

probability to encounter another active node and, hence, to develop a link that, in turn, increases the effective number of partnerships. In Fig. 3c we have plotted the infection prevalence in the metastable state as a function of p_2 (probability of being active). We can see that, for high values of the probability p_2, the prevalence curves for $\gamma_1 = 0.1$ and $\gamma_1 = 3$ with the same value of $\beta = 0.05$ are almost similar to each other, while for lower values of p_2 they are different. In fact, since p_2 only depends on the ratio of γ_1 and γ_2, for a same value of p_2 the duration of links for $\gamma_1 = 0.1$ is 30 times higher than that of $\gamma_1 = 3$. In this figure, we can see that this difference between the values of the link duration is only significant for low values of p_2.

To test the relevance of the threshold β^* obtained from the N-intertwined equations in section 2, we performed another set of simulations where for each node we assigned random values to γ_1 and γ_2. Next, we used the $B^\star$ matrix in equation (5) to find the threshold value β^* for the transmission rate. Since the N-intertwined equations give an upper bound for the nodal infection probabilities in the exact process, if the transmission rate β is lower than β^* we expect that the infection in the exact process die out. In Fig. 4, we have plotted the result obtained from simulating the exact process for different configurations of parameters. In all the simulations, the transmission rate β is slightly lower than the threshold value β^* and we can see the infection is dying out.

References

1. Sternberg, S. *Dynamical systems* (Courier Corporation, 2010).
2. Pastor-Satorras, R. & Vespignani, A. Epidemic spreading in scale-free networks. *Phys. review letters* **86**, 3200 (2001).
3. Juher, D., Saldaña, J., Kohn, R., Bernstein, K. & Scoglio, C. Network-centric interventions to contain the syphilis epidemic in san francisco. *Sci. Reports* **7**, 6464 (2017).
